# Response of Vegetation and Soil Characteristics to Grazing Disturbance in Mountain Meadows and Temperate Typical Steppe in the Arid Regions of Central Asian, Xinjiang

**DOI:** 10.3390/ijerph17124572

**Published:** 2020-06-25

**Authors:** Xu Bi, Bo Li, Xiangchao Xu, Lixin Zhang

**Affiliations:** 1 College of Resources and Environment, Shanxi University of Finance and Economics, Taiyuan 030006, China; 2 Faculty of Geographical Science, Beijing Normal University, Beijing 100875, China; 3 Institute of Science & Technology Information of Shanxi, Taiyuan 030024, China; iostreamatlab@gmail.com; 4 College of Urban and Environmental Science, Peking University, Beijing 100871, China; daxin897@126.com

**Keywords:** grazing, mountain meadow, temperate typical steppe, soil physicochemical properties, vegetation nutrients

## Abstract

Grazing is one of the most common causes of grassland degradation, therefore, an assessment of soil physicochemical properties and plant nutrients under grazing is important for understanding its influences on ecosystem nutrient cycling and for formulating appropriate management strategies. However, the effects of grazing on grassland soil physicochemical properties and plant nutrients in mountain meadow and temperate typical steppe in the arid regions are still unclear. Therefore, we investigated the vegetation nutrient concentrations of nitrogen, phosphorus and potassium (N, P, and K) as well as soil physicochemical properties in the topmost 40 cm depth soil, to evaluate how these factors respond to grazing disturbance in a mountain meadow and temperate typical steppe within a mountain basin system in arid regions. Our results revealed that the soil bulk density values at depth of 0–40 cm increased after grazing in the mountain meadow and temperate typical steppe, whereas the soil water content decreased in the mountain meadow and increased in the temperate typical steppe after grazing. In the mountain meadow, soil total N and available P in addition to vegetation N and P concentrations increased in response to high-intensity grazing, while soil available N, available K and vegetation K decreased after grazing; in addition, soil pH, soil total P and K showed no significant changes. In the temperate typical steppe, the soil total P, soil available N, P, and K, and vegetation N, P, and K increased under relatively low-intensity grazing, whereas soil pH and soil total K showed no significant changes except for the deceasing soil total N. Our findings showed the different responses of different grassland ecosystems to grazing. Moreover, we propose that further related studies are necessary to better understand the effects of grazing on grassland ecosystems, and thereby provide a theoretical basis for the sustainable use of animal husbandry and ecological restoration of grasslands.

## 1. Introduction

Grasslands covering about one-third of the global terrestrial area [1] not only provide resources for animals and plants but play an essential role in primary production, water conservation, ecosystem diversity, and other ecosystem services [2]. However, recent studies have indicated that approximately 90% of the grasslands in Northern China have degraded to a certain extent in Northern China [3]. The physical, chemical, and biological properties of soil worsen as soil degrades, further causing a decrease in ecosystem productivity [4]. Grazing is one of the primary modes of utilizing grasslands, whose patterns and intensity affect the physicochemical properties of soil and vegetation, representing the health status of grassland ecosystems to some extent [5]. Livestock grazing is considered to be the most important factor affects soil physicochemical properties [6]. These characteristics vary greatly with the types of grassland and soil, which in turn affects the absorption of water and nutrients by plants [7]. For example, soil chemical properties can affect the decomposition of vegetation and animal residues by microorganisms, thus further affecting the absorption of vegetation nutrients [8]. In addition, soil available nutrients are of vital importance to animal husbandry playing an important role in improving grassland productivity [9]. Therefore, research on soil degradation should focus on the changes in soil physical and chemical properties and their responses to grazing [10]. The influences of grazing on the physicochemical properties of grassland soil can indicate the ecological consequences of grazing and clarify soil degradation mechanisms responsive to overgrazing.

To explore the effect of grazing on soil properties in grassland ecosystems, many experiments have been conducted; however, the obtained results were contradictory. The effects of grazing on the soil environment, such as soil physicochemical properties, vary greatly among different studies [11,12]. Inappropriate grazing can result in an increase in soil compaction and a decrease in soil quality [13]. As indicated by previous studies [14], grazing increases soil organic carbon (C), total N, total P, and available P and K, while other researches have noted that grazing reduced these soil indicators [15,16,17]. In addition, some researchers have noted that grazing has no effect on soil nutrients [18,19].

The distribution of mountains and basins follows a typical geomorphic structure in the arid region of Central Asia, and is defined as the mountain basin system (MBS) by Zhang [20]. Grassland is the major form of vegetation in the MBS in Central Asia, and livestock grazing is the main use pattern of grassland in this region. In early studies, the effects of grazing on plants have mostly been explored in terms of plant diversity [21], plant coverage [22,23], plant productivity [24], and plant C and N storage [25] in the MBSs. However, the effects of livestock trampling on vegetation nutrient concentrations and soil physicochemical properties are rarely documented, compared with the above indicators. In addition, many current studies are limited to a single grass type, and the responses of vegetation nutrient concentrations and soil physicochemical properties to grazing in different grassland types in MBS are poorly studied. The purpose of our research was to explore the physicochemical properties of soil and vegetation nutrient concentrations under different grazing management practices in a summer pasture and spring/autumn pasture in an MBS of Central Asia. Our guiding hypothesis in this study was that grazing would change the nutrient cycle between soil and vegetation. In addition, grazing would make the soil more compacted. Soil water content, soil pH, soil chemical properties concentrations (total N, P, K, and available N, P, K), and vegetation nutrient concentrations of N, P, and K would either increase or decrease in response to grazing. With different geographical conditions of the mountain meadow (MM) and temperate typical steppe (TTS), we hypothesized that vegetation nutrient concentrations and soil physicochemical properties have different responses to grazing.

## 2. Materials and Methods

### 2.1. Study Area

**Figure 1:**
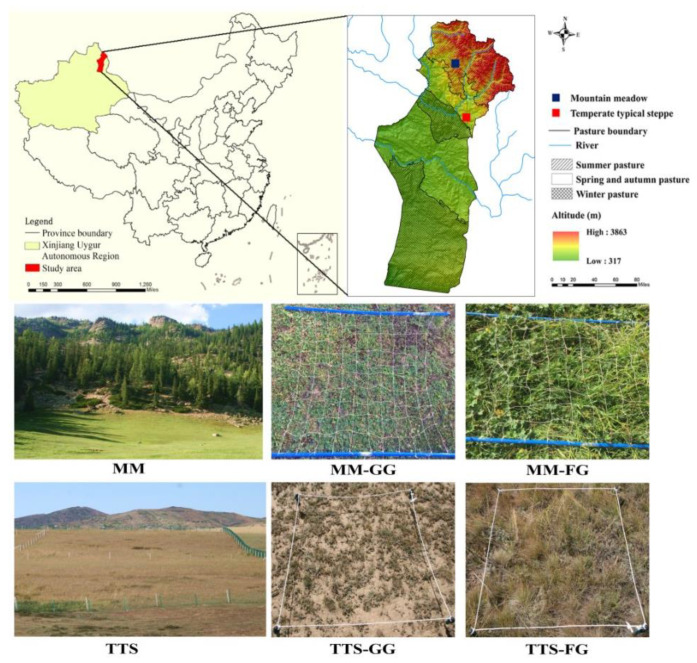
Location map of the study area and sample plots. MM: mountain meadow; TTS: temperate typical steppe; GG: grazed grassland; and FG: fenced grassland.

Fuyun County is a representative MBS in Central Asia, located in the northeastern edge of the Xinjiang Uygur Autonomous Region of China (Figure 1). The study area lies between the southern margin of the Altai Mountains and the northern Junggar Basin, covering an area of 32,186 km^2^ with an elevation of 317–3863 m. This area has a continental cold temperate arid climate, characterized by the mean daily temperature of −17.5 °C and 24.5 °C in the coldest and warmest months, respectively. The average annual precipitation was 208 mm, with precipitation decreasing from south to north of the study area along the elevation gradient. Grassland is the main land cover type in this area, covering almost 90% of the whole area. Fuyun County contains ten types of grasslands [26], transitioning from desert to alpine meadows. Grasslands are used mainly for grazing (mostly cattle and sheep), divided into summer, spring/autumn, and winter pastures according to the time when grasslands are utilized. Overgrazing is a prominent problem, especially in spring and autumn pastures. In this study, we chose a MM (representative of summer pasture) and a TTS (representative of spring and autumn pasture) to explore the effects of grazing on grassland vegetation nutrient concentrations and soil physicochemical properties.

### 2.2. Field Sampling and Laboratory Analysis

#### 2.2.1. Experimental Design

Two grassland types were selected: MM and TTS, which were enclosed in 2006 and 2011, respectively. Each grassland type has a fenced grassland (FG) area and a free-grazing grassland (GG) area. There were no human interference measures in the fenced area, and the free grazing area was seasonal grazing. MM and TTS was utilized from June to the end of August and in spring and autumn each year, respectively (Table 1). Plant and soil samples were taken in August 2017 when the aboveground biomass peaked. Four 10 × 10 m^2^ sampling plots (500–1000 m spacing) were investigated in the fenced area and grazing area of two grass types.

In each plot, three quadrats of 100 cm × 100 cm were set up to investigate vegetation properties. The aboveground part was cut and stored by different species in envelopes. The underground biomass was obtained using a diameter root auger (9 cm), with sampling depths of 0–10, 10–20, and 20–40 cm (the root biomass below 40 cm was very low and was ignored). Soil, stones, and other debris in the vegetation sample were washed and removed. The aboveground parts of the collected vegetation were oven-dried at 65 °C for 48 h to constant weight and weighed. In each sample area, three soil profiles were selected based on the S-shaped pattern to collect soil profile samples using a cutting ring (100 cm^3^ volume). Soil samples were assembled at depths of 0–10, 10–20, and 20–40 cm. Plant roots and stones were removed from the soil samples. After drying, the soil samples were sieved through a 0.14 mm mesh and stored for future testing.

The functional groups of MM were composed of leguminous, gramineous, sedge, and forbs species. The plant dominant species were *Trifolium incarnatum, Alchemilla pinguis,* and *Poa angustifolia* (Table 2). The grazing intensity of MM was relatively low with a grazing pressure index of 1.73. In the MM, lower plant coverage, average height, diversity index, and richness index were found in the grazing GG sites than in the FG sites [27]. In terms of vegetation productivity, the aboveground biomass of the GG sites was significantly lower than that of the FG sites, while the underground biomass was markedly higher than that of the FG sites (Table 2).

The functional groups of TTS were composed of leguminous, gramineous, sedge, semi-shrubs, and forbs species. The plant dominant species of the TTS were *Artemisia frigida, Kochia prostrata*, and *Festuca ovina* (Table 2). The grazing intensity of the TTS was greater than that of the MM with a grazing pressure index of 3.89. A previous study showed that in the TTS, the average height and coverage of plant at the GG sites were lower than those at the FG sites, while the diversity index and richness index were higher at the FG sites than at the GG sites [27]. The aboveground biomass and underground biomass of the GG plot were significantly lower than those of the FG plot in the TTS (Table 2) [27].

#### 2.2.2. Measures indicators

C concentration in the plants and soils was measured by a Multi N/C 3100 TOC-Analyser produced by Germany, and N concentration was determined using modified Kjeldahl method. P and K concentrations in the plants and soils were measured via the Mo-Sb calorimetric method and NaOH fusion-flame photometric method [28], respectively. The oven-dried soil was used to measure the bulk density (BD), and soil water content (SWC) was detected by the gravimetrically method at 105 °C for 24 h. The soil pH (pH) was measured with a pH metre at a soil-water ratio of 1:2.5 (w:v). The soil available N (AN) concentration was measured by the alkali diffusion method. After being dissolved by sodium hydroxide (NaOH, 1.8 mol·L^−1^), the available nitrogen in soils was converted to ammonia, and then absorbed by boric acid (H_3_BO_3_). The spectrophotometer method was applied to measure the soil available P and soil available K after being extracted with sodium bicarbonate (NaHCO_3_, 0.5 mol·L^−1^) and ammonium acetate (NH_4_OAc, 1 mol·L^−1^), respectively [28].

### 2.3. Data Processing

Through a normality test, the data followed a normal distribution. One-way analysis of variance (ANOVA) was conducted for the differences among the three soil layers and the two management measures in the grassland using IBM SPSS Statistics (ver. 20.0; IBM, New York, NY, USA), with *p* ≤ 0.05 being considered statistically significant. The least significant difference (LSD) post hoc test was used to multiple comparisons when significance was observed.

## 3. Results

### 3.1. Soil Physical Properties

**Figure 2:**
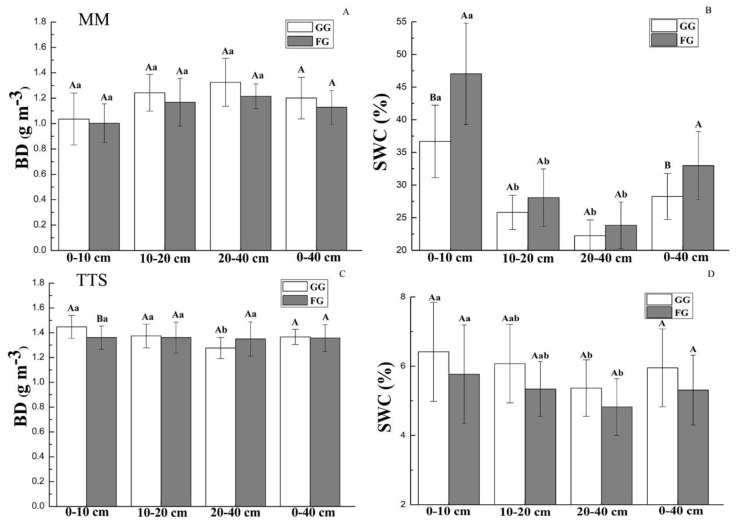
Soil physical properties in the MM and TTS under the grazing and fencing treatments. (**A**): Soil bulk density in MM; (**B**): Soil water content in MM; (**C**): Soil bulk density in TTS; (**D**): Soil water content in TTS. Values are the means ± standard deviation, the bar charts marked with different letters on the top of the column (Different lowercase letters for different soil layers, and different capital letters for different treatments) are significantly different (*p* < 0.05). MM: mountain meadow; TTS: temperate typical steppe; GG: grazed grassland; FG: fenced grassland. BD: bulk density; and SWC: soil water content.

In comparison to the FG sites, the GG sites were associated with increases in soil BD in the MM of all the investigated soil layers and in the 0–10 cm and 10–20 cm soil depths in the TTS (Figure 2A,C). With the increase of soil depth, the BD increased in MM, while decreased in TTS. The BD in 0–40 cm soil layer was slightly higher at the GG sites (1.20 ± 0.16 g·m^−3^) than at the FG sites (1.12 ± 0.13 g·m^−3^) in the MM, and the BD in 0–40 cm soil layer at the FG sites and GG sites in TTS were 1.36 ± 0.06 g·m^−3^ and 1.35 ± 0.10 g·m^−3^, respectively.

In both the MM and TTS, the SWC values decreased with increasing soil depth at the FG and GG sites. (Figure 2B,D). In the MM, the topsoil had the highest SWC values in all the surveyed soil layers, and significantly higher than other soil layers. The SWC values decreased from 36.68% to 22.23% and from 47.02% to 23.82% from 0–10 cm to 20–40 cm at the GG and FG sites, respectively. The SWC values reduced in all surveyed soil layers in the MM after grazing. Compared to that at the FG sites, the SWC values at the GG sites decreased by 21.99%, 7.99%, 6.66%, and 14.33% at 0–10, 10–20, 20–40, and 0–40 cm soil layers, respectively (Figure 2B).

In the TTS, the SWC values decreased from 6.41% to 5.36% and from 5.76% to 4.82% from the 0–10 to 20–40 cm soil depths in the GG and FG sites, respectively. However, the SWC values in each soil layer increased after grazing in the TTS. The GG sites showed SWC values 1.11, 1.14, 1.11, and 1.12 times greater than FG sites, at 0–10, 10–20, 20–40, and 0–40 cm soil layers, respectively (Figure 2D).

### 3.2. Soil Chemical Properties

**Figure 3:**
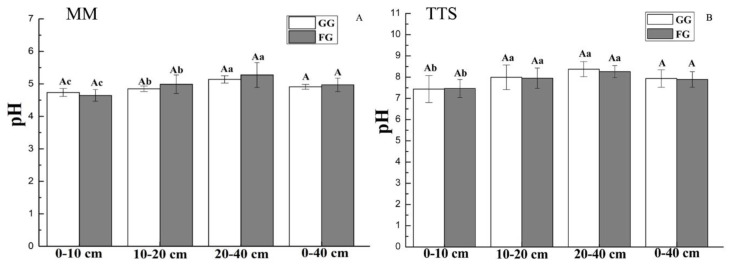
(**A**): Soil pH in the MM under the grazing and fencing treatments; (**B**): Soil pH in the TTS under the grazing and fencing treatments. Values are the means ± standard deviation, the bar charts marked with different letters on the top of the column (Different lowercase letters for different soil layers, and different capital letters for different treatments) are significantly different (*p* < 0.05). MM: mountain meadow; TTS: temperate typical steppe; GG: grazed grassland; FG: fenced grassland.

**Figure 4:**
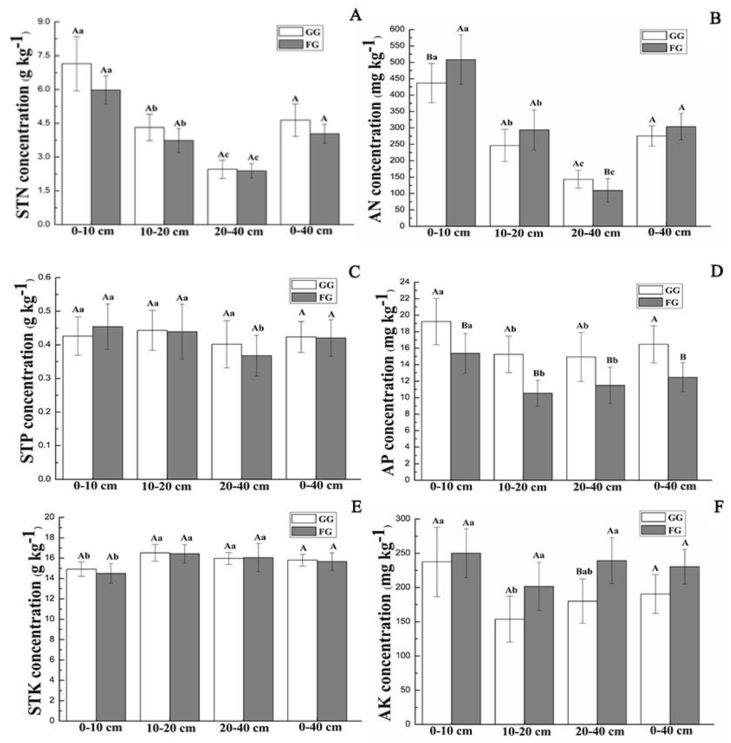
Soil chemical properties under the grazing and fencing treatments in the MM. (**A**): Soil total N concentration; (**B**): Soil available N concentration; (**C**): Soil total P concentration; (**D**): Soil available P concentration; (**E**): Soil total K concentration; (**F**): Soil available K concentration. Values are the means ± standard deviation, the bar charts marked with different letters on the top of the column (Different lowercase letters for different soil layers, and different capital letters for different treatments) are significantly different (*p* < 0.05). MM: mountain meadow; GG: grazed grassland; FG: fenced grassland; STN: soil total N; STP: soil total P; STK: soil total K; AN: soil available N; AP: soil available P; and AK: soil available K. The same below.

The soil chemical properties under grazing and fencing treatments in the MM and TTS are shown in Figure 3 and Figure 4. 

Soil pH values increased with increasing soil depth, showing no significant difference between the GG plot and FG plot in all surveyed soil layers in MM and TTS (Figure 3). As for the MM with acidic soil, the soil pH values in the 0–40 cm soil depth ranged from 4.73 to 5.13 and from 4.64 to 5.27 at the GG sites and FG sites, respectively (Figure 3A); while in the TTS with alkaline soil, the soil pH values in the 0–40 cm soil layers ranged from 7.43 to 8.37 and from 7.46 to 8.25 at the GG and FG sites, respectively (Figure 3B).

The soil properties under different treatments in the MM are shown in Figure 4. The concentrations of soil total N, P, and K did not show significant differences between the grazing and fencing sites. Grazing increased the soil total N concentrations in all surveyed soil depths in the MM, and the soil total N concentrations decreased with increasing soil depth at both the GG and FG sites (Figure 4A). The soil total N concentrations at the GG sites decreased by 19.51%, 15.45%, 2.93%, and 14.99% at 0–10, 10–20, 20–40, and 0–40 cm soil layers, compared to those at the FG sites, respectively (Figure 4A). The soil total K and P concentrations showed no significant difference among the three soil layers at all the investigated sites except for the STP concentration of the 20–40 cm depth at the FG sites (Figure 4C,E *p* > 0.05). The soil total P and K concentrations ranged from 0.36 ± 0.06 to 0.45 ± 0.06 g·kg^−1^ and 14.05 ± 0.96 to 16.51 ± 0.82 g·kg^−1^ in the MM, respectively (Figure 4C,E). Grazing reduced the concentrations of soil available N and K, exhibiting increased soil available P concentrations in all investigated soil layers in the MM (Figure 4B,D,F). The average concentrations of soil available N, P, and K in the 0–40 cm depth at the GG sites were 0.91, 1.32, and 0.82 times those at the FG sites, respectively (Figure 4B,D,F).

**Figure 5:**
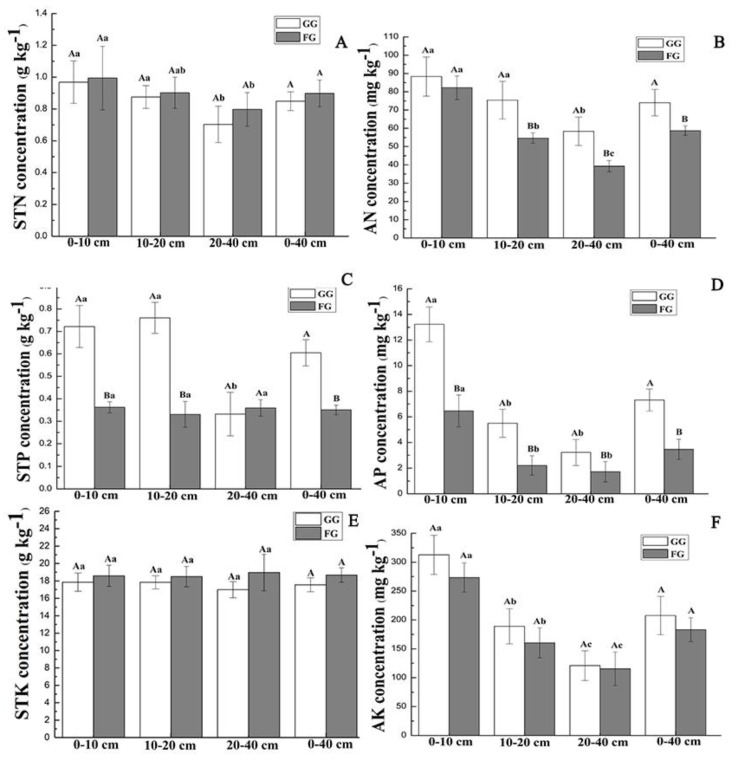
Soil chemical properties under the grazing and fencing treatments in the TTS. (**A**): Soil total N concentration; (**B**): Soil available N concentration; (**C**): Soil total P concentration; (**D**): Soil available P concentration; (**E**): Soil total K concentration; (**F**): Soil available K concentration. Values are the means ± standard deviation, the bar charts marked with different letters on the top of the column (Different lowercase letters for different soil layers, and different capital letters for different treatments) are significantly different (*p* < 0.05). TTS: temperate typical steppe; GG: grazed grassland; FG: fenced grassland.

In the TTS, compared to the FG sites, the GG sites had soil total N concentrations with a reduction of 2.58%, 2.90%, 11.77%, and 5.42% in the soil depths of 0–10, 10–20, 20–40, and 0–40 cm, respectively (Figure 5A). In comparison to the FG sites, the GG sites had 1.99, 2.29, 0.92, and 1.72 times the soil total P concentrations in soil layers of 0–10, 10–20, 20–40, and 0–40 cm, respectively (Figure 5C), and the corresponding values were 0.96, 0.96, 0.89, and 0.93 times as high as the soil total K concentrations, respectively (Figure 5E). Grazing increased the soil available N, P, and K concentrations in each of the investigated soil layers (Figure 5B,D,F). The mean concentrations of soil available N, P, and K at depths of 0–40 cm increased by 26.11%, 111.05%, and 13.37%, respectively (Figure 5 B,D,F).

### 3.3. Vegetation Nutrients

**Figure 6:**
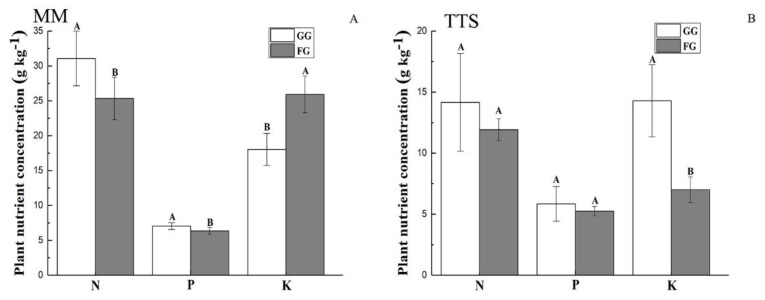
Plant nutrient concentrations in the MM and TTS under the grazing and fencing treatments. (**A**): Plant N, P and K concentrations under the grazing and fencing treatments in MM; (**B**): Plant N, P and K concentrations under the grazing and fencing treatments in TTS. Values are the means ± standard deviation, the bar charts marked with different capital letters on the top of the column are significantly different (*p* < 0.05). MM: mountain meadow; TTS: temperate typical steppe; GG: grazed grassland; FG: fenced grassland.

In the MM, in comparison to the FG sites, the sites with grazing activities resulted in markedly increased plant N and P concentrations (by 22.59% and 10.72%, respectively) and a decreased K concentration (30.37%) (Figure 6A, *p* < 0.05). Grazing led to a marginal increase in the plant N and P concentrations in the TTS (Figure 6B, *p* > 0.05), where the plant K concentration improved significantly after grazing (Figure 6B, *p* < 0.05). Plant N, P, and K increased by 18.73%, 11.49%, and 104.16% after grazing, respectively, at the GG sites compared with that at the FG sites (Figure 6B).

## 4. Discussion

### 4.1. Effects of Grazing on Soil Physical Properties

Grazing is one of the most significant factors affecting soil properties [29]. The effects of grazing on soil compaction are inconsistent [30]. Livestock trampling on a grassland might result in soil compaction and a decrease in soil porosity, increasing BD, reducing water infiltration, aggregate stability, and vegetation cover, and resulting in changes in soil physical properties [31]. 

Our study indicated that grazing resulted in the grassland soil experiencing increased compaction and increased average BD values at depths of 0–40 cm in both the MM and TTS (Figure 2A,C), as in accordance with the previous studies [32,33]. Due to the mechanical stress imposed on the soil, livestock trampling had a strong effect on soil compaction, generally contributing to an increase in BD after grazing [34]. In addition, the increase of plant roots and soil microorganisms may have reduced the BD at the FG sites [35]. In comparison to the TTS, the MM has a higher elevation with abundant precipitation and a thick soil humus layer [27]. At the FG and GG sites of the MM, as the depth increased, the soil BD tended to increase (Figure 2A), indicating that surface soil development was better than deeper soil development. The root system of the plants likely increased the organic matter of the surface soil, increasing the porosity of the surface soil and reduce the BD [36]. However, the BD of the soil surface was higher than that at the lower soil depths in the TTS (Figure 2C). The TTS has a higher grazing intensity with less precipitation and soil organic matter. With the increase in animal trampling, the spatial pattern of the soil porosity distribution changed, resulting in a decrease in the soil porosity of the topsoil and an increase in the soil BD [37].

Grazing reduced the SWC values of all surveyed soil depths in the MM (Figure 2B), consistent with the findings of Chai et al. [38] that the soil moisture in the fenced area was higher than that outside the fenced area when a large amount of precipitation occurred. This scenario occurred because the grass density, coverage, height, and biomass of the fencing grassland area were obviously greater than those factors outside the fenced area, and therefore, the fenced area had a significant interception effect on precipitation. In contrast, in the grazed grassland, the vegetation coverage was reduced due to the large amount of grazing by livestock, impeding the interception of precipitation. In addition, the increased soil compaction decreases the soil macroporosity, and thus, reduced SWC in grazed grassland. Greater SWC values were observed at the GG sites in all surveyed soil layers in the TTS (Figure 2D). The low-altitude TTS has low levels of precipitation; as precipitation decreases, the soil gradually dries out, and evaporation continues to increase as the temperature rises, causing changes in the SWC inside and outside the fenced area. However, this change in the grazing grassland was affected by livestock trampling. Livestock trampling increased the soil density and decreased the porosity of a grassland, leading to less evaporation and higher SWC values of the soil at the GG sites than FG sites.

### 4.2. Effect of Grazing on Soil Chemical Properties

Grassland vegetation nutrients are mainly derived from soil, whose nutrients can influence the growth of grassland vegetation directly or indirectly [39]. The change in soil chemical properties is a complicated process, being significantly affected by grazing. Livestock trampling reduces the biomass and height of vegetation, resulting in soil surface exposure and erosion [40], may resulting in a drop in soil nutrients [39].

Previous research has reported that soil pH tends to increase after grazing [41,42], while Tang et al., [33] found the highest pH under seasonal grazing. However, our study showed no significant changes in pH in the investigated soil layers in the TTS and MM (Figure 3), indicating that grazing had a limited effect on soil pH. The soil pH values in the MM were acidic and significantly lower than those in the TTS (Figure 3). Strong root exudates and soil respiration produce relatively more carbonic acid and organic acid, as well as higher local precipitation, which may have accounted for the lower pH in the MM than in the TTS (Figure 3) [27,42].

In our study, grazing had the opposite effect on fixing soil carbon and nitrogen levels in the MM and TTS, probably due to differences in grazing intensity, grassland type, and climatic factors [43]. Soil C and N inputs are mainly derived from vegetation, litter, root turnover, and animal and plant exudates [42]. The MM and TTS are located at different altitudes with climatic conditions varying. Located at a relatively high elevation (2166 m), the MM is a summer pasture with relatively abundant rainfall, fine forage, and lower grazing pressure. The TTS is a spring and autumn pasture at a low altitude (1414 m) (Table 1). Compared with the MM, the TTS has less precipitation and higher temperature, resulting in a significantly lower vegetation coverage (only 35%) at the GG sites than the FG sites (Table 2). In the MM, the soil total N increased after grazing (Figure 4A), as influenced by high vegetation coverage at the GG and FG sites in the MM (>85%) (Table 2), the root mass of vegetation as the main source of soil carbon and nitrogen [44], and grazing-related growth of dense root vegetation (*Astragalus membranaceus* and *Carex liparocarpos*) (Table S1) [27]. Therefore, the increased belowground biomass of the vegetation at the GG sites was responsible for the increase in the soil total N. In addition, livestock trampling and grazing can promote the growth and mortality of roots, and the amount of litter that enters the soil [45], thereby accelerating the rate of root turnover and litter decomposition [46]. Grazing can facilitate the accumulation of vegetation productivity to the root system, thus increasing the amount of N that inputs to the soil. Therefore, N input from plant roots into the soil will increase after grazing [47]. In contrast, the soil total N of the GG sites decreased in all investigated soil layers in the TTS (Figure 5A), which was associate with grazing-driven reduction of the aboveground and belowground vegetation biomass and thus reduced the input of total nitrogen in soil [27,48].

Moreover, the low vegetation coverage at GG sites enhanced soil mineralization, nitrification, and ammonization, compared to fencing grassland. As a result, soil respiration increased, and N_2_O emissions increased [49]. Therefore, the decreased vegetation coverage at the GG sites increased the area directly influenced by wind erosion on the soil surface, increasing the loss of soil organic C and soil total N from soil respiration and mineralization [50].

The soil total P showed no significant difference between the FG and GG sites at all the layers in the MM (Figure 4C), which was in agreement with the results of Li et al. [51], who conducted an experiment in an alpine meadow on the Qinghai-Tibetan Plateau. Our observations indicated that soil total P was insensitive to grazing in the MM, concurring with the findings of Rui et al. [52]. The solubility of P in soil was low, and soil P mainly comes from rock weathering rather than organic matter decomposition, causing a higher stability of soil P [53]. Therefore, the soil total P was minimally affected by the amount of organic matter derived from vegetation as well as from grazing. In contrast, the soil total P concentration significantly increased after grazing in the TTS (Figure 5C). Zhang et al. [54] noted that grazing reduces fungal activity, thus limiting the loss of P. Mycorrhizal fungi are crucial players in effective symbiosis, enabling plant to get soil P from other forms that are not available [55]. Intensive grazing could inhibit the uptake of phosphorus by plant, thus helping to maintain soil phosphorus levels relatively stable. Therefore, the soil total P concentration at the GG sites was higher than that in the FG sites due to the high grazing intensity. There was no significant difference in soil total K concentrations between the FG and GG sites both in the MM and TTS (Figure 4E and Figure 5E) because soil total K was relatively adequate and did not limit plant growth [47]. The changes in species composition and biomass after grazing had little effect on microbial mineralization and soil nutrient elements, agreeing with the work of Shi et al. in alpine grasslands [47].

Concentrations of available nutrients in soil are related to soil mineralization, plant absorption, and livestock waste [56]. In a light grazing environment, plant has a strong influence on the absorption and utilization of soil available nutrients. In comparison to the TTS, the MM had lower grazing intensity, resulting in lower concentrations of soil available N and K in the soil (Figure 4B,F) because of the absorption and utilization of available N and K by the plants [14]. Soil organic P cannot be directly absorbed by plants, and most plants need soil organic P to be converted into inorganic P that can be absorbed and used by plants through mineralization. Higher belowground biomass was recorded for the GG sites than for the FG sites in the MM. The BGB can be increased after grazing by promoting the growth of leguminous species and sedge species with dense root systems [27]. Plant root microorganisms can increase the soil organic P mineralization and inorganic P accumulation rate [57], resulting in the higher soil available P at the GG sites that at the FG sites in the MM (Figure 4D). However, using soil available nutrients under a high grazing intensity is not effective, favoring the accumulation of soil available N, P, and K in the soil at the GG sites in TTS (Figure 5B,D,F). In our study, grazing markedly reduced vegetation coverage and enhanced the turf soil in the air at the GG sites, therefore increasing the decomposition and erosion of soil [58]. Moreover, livestock trampling on a grassland could return litter into the soil and increase microbial mineralization [59]. In addition, the release from livestock grazing and trampling could protect the surface of the soil structure and avoid excretion of herbivores (e.g., dung and urine), decreasing microbial processes and soil mineralization [60]. Therefore, grazing may promote decomposition and the uptake rate of total soil nutrient elements into available nutrients [61]. Furthermore, in our study, the increased soil available N, P, and K concentrations were partly attributed to less demand for soil available nutrients due to decreased biomass production after grazing in the TTS.

### 4.3. Effects of Grazing on Plant Properties

As essential nutrients for plant growth, N, P, and K participate in the structural composition and functional operation of cells and play important roles in the growth and development of plants, the composition of communities, and the structure and function of ecosystems [62]. K also plays an important role in the physiological and biochemical processes of plants and is closely related to the drought and disease resistance of plants [63]. Nevertheless, research on it is rare compared with research on C, N, P, and other elements.

Grazing made a significant influence on the N and P concentrations of vegetation in the MM. The vegetation N and P concentrations were higher at the GG sites than at the FG sites (Figure 6A). Under light grazing, the plants had overcompensated growth, and the nutrient absorption rate was fast. Moreover, the grazing livestock grazed on the grass removed part of the mature tissue from the plant surface, and the remaining young tissue contained a high nutrient concentration [64]. At the same time, grazing increased root exudate and promoted microbial activities, leading to an increase in available nutrients provided by the soil to the plants, thus changing the chemometrics of the plant communities [60]. Moreover, in the MM, grazing markedly increased the relative coverage and biomass of leguminous species with dense root systems and strong N fixation ability [27]. 

Soil is an important source for plants to obtain nutrients, and the content of soil nutrients is directly linked to the growth status and yield of plants. Generally, the level of soil available nutrients determines the amount of vegetation nutrients. Therefore, the change trends in plant nutrient concentrations (N, P, and K) after grazing were consistent with the change trends in soil available N, P, and K at all the sites in the MM and TTS (Figure 4, Figure 5 and Figure 6), except for plant N in the MM. 

## 5. Conclusions

This study revealed the effects of livestock grazing on plant nutrient concentrations and soil physicochemical properties within 0–40 cm soil depths in a mountain meadow and temperate typical steppe within a mountain basin system of Central Asia. We found that plant nutrient concentrations and soil physicochemical properties in MM and TTS responded differently to both grazing and soil depth. In terms of the physical properties of the soil, our results indicated that grazing compacted the surface soil in both the MM and TTS, and SWC decreased in MM but increased in TTS after grazing. For the chemical properties of the soil, in the MM, the soil total N and soil available P of the soil increased in response to grazing, while the soil available N and K decreased; the pH, soil total P and K showed no significant change after low-intensity grazing. In the TTS, the soil total P and soil available nutrients (N, P, and K) increased after relatively high-intensity grazing, while soil total N decreased. At the same time, we found that grazing had no significant effect on soil pH and soil total K in the TTS. In addition, vegetation nutrients (N, P, and K) increased after grazing at all investigated sample sites except for plant K in the MM. 

These results suggest that grazing or trampling have had a serious impact on the MM and TTS in a mountain basin system. This study well predicted grassland degradation, being conducive for managers to respond in a timely manner to implement protection and recovery measures in mountain meadows and temperate typical steppe within a mountain basin system in arid regions. In addition, in order to get a deep understanding of the effect of grazing on grassland plant and soil properties, it would be desirable to conduct an experiment to explore the effect of different grazing intensities on the physicochemical properties of plant and soil, as this would deepen our understanding of ecosystem feedback to grazing disturbance and provides important essential data to guide the appropriate use of grassland resources and maintain the health of grassland ecosystems.

## Figures and Tables

**Table 1:** The information of the plots.

Sample Plots	Longitude (°)	Latitude (°)	Elevation (m)	MAP (mm)	MAT (°C)	Agrotype	Utilization Period	GPI
Mountain meadow	89.41	47.57	2066	359.42	−1.71	Mountain meadow soil	Summer	1.73
Temperate typical steppe	89.75	46.97	1414	277.06	2.23	Chestnut soil	Spring/autumn	3.89

Note: MAP: mean annual precipitation; MAT: mean annual temperature; GPI: grazing pressure index.

**Table 2:** The functional group and effects of grazing on the plant coverage and biomass in MM and TTS [27].

Grassland Type	Functional Group	Treatments	Coverage (%)	Above/Belowground Biomass (g·m^−2^)	Dominant Species
Mountain meadow	Leguminous species Gramineous Species Sedge species Forbs species	FG	95 ± 3.83A	282.5 ± 26.5A (Above) 976.50 ± 67.59B (Below)	*Poa angustifolia,* *Alchemilla pinguis*
		GG	85.5 ± 5.26B	65.9 ± 9.51B (Above) 1193.75 ± 90.40A (Below)	*Trifolium incarnatum,* *Alchemilla pinguis*
Temperate typical steppe	Leguminous species Gramineous species Sedge species Semi-shrubs species Forbs species	FG	68.75 ± 4.79A	144 ± 4.71A (Above) 1090.68 ± 84.27A (Below)	*Artemisia frigida,* *Festuca ovina*
		GG	35 ± 4.08B	64.55 ± 2.83B (Above) 564.07 ± 41.69B (Below)	*Artemisia frigida,* *Kochia prostrata*

Note: values are the means ± SE and the difference are significant between the grazing treatment and the enclosure treatment when followed by different capital letters (*p* < 0.05). GG: grazed grassland; and FG: fenced grassland.

## Supplementary Materials

The following are available online at https://www.mdpi.com/1660-4601/17/12/4572/s1, Table S1: Effects of grazing on the aboveground biomass of different functional groups and species in MM and TTS.

## Abbreviations

MM: mountain meadow; TTS: temperate typical steppe; GG: grazed grassland; FG: fenced grassland; BD: bulk density; SWC: soil water content; N: nitrogen; P: phosphorus; K: potassium; STN: soil total N; STP: soil total P; STK: soil total K; AN: soil available N; AP: soil available P; AK: soil available K.
