# Supplementary material for: Response of Vegetation and Soil Characteristics to Grazing Disturbance in Mountain Meadows and Temperate Typical Steppe in the Arid Regions of Central Asian, Xinjiang
Source: Int J Environ Res Public Health. 2020 Jun 25;17(12):4572. doi: 10.3390/ijerph17124572 (PMC7345459; doi:10.3390/ijerph17124572)
Supplement: Supplementary file 1 [file ijerph-17-04572-s001.pdf]

Table S1 Effects of grazing on the aboveground biomass of different functional groups and species in MM and TTS.

Functional Group	GG			FG		
	AGB of Species (g·m^{-2})	AGB of Group (g·m^{-2})	Proportion (%)	AGB of Species (g·m^{-2})	AGB of Group (g·m^{-2})	Proportion (%)
MM						
Leguminous species	*Trifolium incarnatum* (9.8 ± 7.09)<br>*Astragalus membranaceus* (1.5 ± 3)	11.3 ± 4.49A	18.02% ± 9.1A	*Trifolium incarnatum* (1.3 ± 1.05)	1.3 ± 1.36B	0.47% ± 0.52B
Gramineous species	*Poa angustifolia* (0.9 ± 1.05)	0.9 ± 1.8B	1.29% ± 2.58B	*Alopecurus aequalis* (13.1 ± 10.56)<br>*Poa angustifolia* (93.2 ± 44.18)<br>*Dactylis glomerata* (2.6 ± 0.95)<br>*Lolium perenne* (4.3 ± 3.32)	113.2 ± 39.81A	39.51% ± 10.6A
Sedge species	*Carex buekii* (6 ± 3.21)	6 ± 2.72A	8.91% ± 2.4A	*Carex buekii* (2.8 ± 2.37)	2.8 ± 2.3A	0.96% ± 0.76B
Forbs species	*Alchemilla pinguis* (44.3 ± 12.05)<br>*Achillea millefolium* (1.2 ± 1.38)<br>*Taraxacum mongolicum* (2.2 ± 3.2)	47.7 ± 10.67B	71.78% ± 6.4A	*Alchemilla pinguis* (160.4 ± 20.07)<br>*Achillea millefolium* (0.4 ± 0.46)<br>*Taraxacum mongolicum* (3.4 ± 2.78)	165.2 ± 21.7A	59.05% ± 10.7A
TTS						
Leguminous species	*Astragalus membranaceus*(2.5 ± 0.53)	2.5 ± 0.53A	3.89 ± 0.95%A	*Astragalus membranaceus* (1.5 ± 2.01)	1.5 ± 2.01A	1.23% ± 1.58B
Gramineous species	*Festuca schinz* (4.58 ± 3.14)<br>*Stipa capillata* (2.23 ± 0.57)<br>*Poa angustifolia* (4.01 ± 8.02)	10.82 ± 6.53B	16.87% ± 10.44B	*Festuca schinz* (60.09 ± 44.38)<br>*Stipa capillata* (22.69 ± 32.62)	82.78 ± 40.55A	55.49 ± 19.41%A
Sedge species	*Carex liparocarpos* (6.2 ± 2.84)	6.2 ± 2.84A	9.71% ± 4.87A	*Carex liparocarpos* (3 ± 0.64)	3 ± 0.64A	2.14% ± 0.53B
Semi-shrubs species	*Artemisia frigida* (29.51 ± 13.27)<br>*Kochia prostrate* (8.71 ± 8.05)	38.22 ± 8.85A	59.27% ± 13.76A	*Artemisia frigida* (38.14 ± 25.48)<br>*Kochia prostrate* (10.43 ± 16.29)	48.57 ± 22.50A	34.03 ± 13.02%B
Forbs species	*Polygonum aviculare* (5.43 ± 7.28)<br>*Chenopodium glaucum* (0.86 ± 0.59)<br>*Heteropappus hispidus* (0.25 ± 0.5)<br>*Trigonella foenum-graecum* (0.27 ± 0.54)	6.81 ± 7.98A	10.24 ± 11.73%A	*Polygonum aviculare* (8.15 ± 10.79)	8.15 ± 10.79A	7.11 ± 9.78%A

Note: values are the mean ± SE and are significantly different ($p < 0.05$) between grazed and fenced treatments when followed by a different capital letter. MM: mountain meadow; TTS: temperate typical steppe; GG: grazed grassland; FG: fenced grassland; AGB: aboveground biomass.
